# Cor Triatriatum Dexter: The Largest Comprehensive Review in the Field on 124 Worldwide Cases (1968–Now)

**DOI:** 10.3390/jcdd13020076

**Published:** 2026-02-03

**Authors:** Pier Paolo Bassareo, Erica Franco, Sophie Duignan, Massimo Chessa, Mariateresa Cascio, Colin Joseph McMahon, Kevin Patrick Walsh, Marco Alfonso Perrone

**Affiliations:** 1 School of Medicine, University College of Dublin, D04 V1W8 Dublin, Ireland; cmcmahon992004@yahoo.com (C.J.M.); kpjwalsh@me.com (K.P.W.); 2 Mater Misericordiae University Hospital, D07 R2WY Dublin, Ireland; 3 Children’s Health Ireland at Crumlin, D12 N512 Dublin, Ireland; sophie_duignan@hotmail.com; 4 Division of Cardiology, Ospedale Civico di Chivasso, 10034 Chivasso, Italy; efranco@aslto4.piemonte.it; 5 Adult Congenital Heart Disease UNIT, Paediatric and Adult Congenital Heart Centre, IRCCS-Policlinico San Donato, San Donato Milanese, 20097 Milan, Italy; massichessa@yahoo.it; 6 School of Medicine, Vita Salute San Raffaele University, 20132 Milan, Italy; 7 Surrey and Sussex Healthcare NHS Trust, Redhill RH1 5RH, UK; mtcascio3@gmail.com; 8 Division of Cardiology and CardioLab, Department of Clinical Sciences and Translational Medicine, University of Rome Tor Vergata, 00133 Rome, Italy; marco.perrone@uniroma2.it; 9 Clinical Pathways and Epidemiology Unit, Bambino Gesù Children’s Hospital, IRCCS, 00165 Rome, Italy

**Keywords:** cor triatriatum dexter, cor triatriatum dextrum, echocardiography, cardiac magnetic resonance imaging, computed tomography, congenital heart disease

## Abstract

Background. Cor triatriatum dexter (CTD) is a rare congenital heart defect where a membrane divides the right atrium into two chambers, resulting from the incomplete regression of the right valve of the sinus venosus. Due to its rarity, only individual case reports and a limited number of case series have been published to date. This study constitutes the most extensive comprehensive review conducted in this area. Eight factors were evaluated: age at diagnosis, sex, clinical presentation, electrocardiographic findings, imaging (ultrasound, CT, or MRI), associated cardiac anomalies, and patient outcomes. Methods. The electronic databases PubMed and Scopus were searched from their inception until 30 October 2025. Only case reports and case series were considered for inclusion. Studies involving foetuses, autopsies, and animals were excluded. The collected data were primarily presented as percentages. Results. One hundred fourteen studies were found encompassing 124 patients. The mean age at diagnosis was 33.3 ± 9.4 years The most common clinical presentations were dyspnoea (44.3%) and cyanosis (29.5%). The most commonly encountered ECG changes were supraventricular tachycardia/atrial flutter/atrial fibrillation (33.3%) and right bundle branch block (22.6%). On chest X-ray, cardiomegaly was noted in 46.5%. CTD was suspected or diagnosed by echocardiography in 95.2% of cases. The diagnosis was confirmed by CT and/or MRI in 34.1% of cases. A concomitant congenital heart defect was found in 67.7%, especially in the form of all kinds of atrial septal defect (38.1%) and of right valvular and right ventricular involvement (20.1%). An outcome was reported in 97/124. Surgical correction was the treatment of choice in 51.6%. Since 1991, a percutaneous approach has been employed in selected cases (5.1%). Conservative management was the treatment of choice in 43.3%. The mortality rate was 8.2%. Discussion. The principal limitation of this systematic review lies in its reliance solely on case reports and small case series, reflecting the absence of large-scale studies on CTD. Nonetheless, it constitutes the most comprehensive analysis available to date.

## 1. Introduction

Cor triatriatum dexter (CTD) represents an exceptionally uncommon congenital heart anomaly in which a membranous structure divides the right atrium into two distinct chambers [1]. This residual membrane may be either fenestrated or obstructive and can variably separate systemic venous return from the tricuspid inflow, leading to a wide spectrum of haemodynamic effects [2].

The term “cor triatriatum dextrum” had first been described by Yater, whereas the first clinical description in the literature was given by Rossall and Caldwell [3,4].

Regarding embryology, this lesion occurs when the Eustachian valve, or right venous valve of the inferior vena cava, is unusually large and causes obstruction to right ventricular filling. During early cardiogenesis, the right horn of the sinus venosus is guarded by two valves, the right and the left venous valves [5,6]. The smaller left valve becomes incorporated in the septum secundum, but the right valve almost completely divides the right atrium into two chambers. This structure normally regresses between the 9th and the 15th week of gestation, as the cephalic portion forms the crista terminalis and the caudal portion develops into the Eustachian valve of the inferior vena cava and the thebesian valve of the coronary sinus. Extensive fenestrations of the right venous valve may result in a weblike Chiari’s network [7]. Any failure in the normal regression of the right valve of the sinus venosus may rarely occur. This can result in remnants of right sinus valve ranging from partial septation of the right atrium by a prominent Eustachian valve to another small atrial septation called Chiari’s network to complete fenestrated or unfenestrated division of the right atrium (CTD) [8]. Remnants of the embryologic sinus venosus valves are not uncommon. However, remnants which are large enough to obstruct the blood flow through the tricuspid valve (CTD) are very rare [9]. In other cases, the right valve of the sinus venosus may form a pendulous windsock-like structure [10]. Depending on the length of the “stalk” and where it is carried by blood flow, the windsock may obstruct the tricuspid orifice [11], right ventricular outflow tract [10], inferior vena cava [4,12] or atrial septal defect [13].

CTD has an estimated incidence of around 0.025% of all congenital heart diseases. However, a large case series reviewing complication during interventional closure of atrial septal defect found that incomplete forms of CTD were not so rare (5.2% of the cases) [14]. Cor triatriatum sinister is way more common than dexter. In this respect, in a previous review about all cases of cor triatriatum sinister and CTD, the ratio between the two congenital abnormalities was 4.9/1 [15].

Given its rarity, the existing literature on CTD predominantly comprises individual case reports and small series, and no large-scale studies are available. Clinical recognition is often delayed or incidental, as the presentation can occur at any age. While some patients remain asymptomatic and are diagnosed incidentally through imaging, others present in infancy or childhood with cyanosis, or later in adulthood with right-sided heart failure, arrhythmias, or thromboembolic events—depending on the degree of obstruction and the presence of associated defects. This broad clinical variability has contributed to inconsistent reporting and heterogeneous management practices across studies [16].

Recent advances in multimodality imaging—including transthoracic and transoesophageal echocardiography, three-dimensional echocardiography, computed tomography, and cardiac magnetic resonance—have enhanced the detection and anatomical characterisation of CTD [17]. Nevertheless, uncertainties persist regarding its natural history, optimal timing and mode of intervention, and the prevalence of coexisting congenital anomalies. A comprehensive and contemporary synthesis of all reported CTD cases—encompassing demographic data, clinical and electrocardiographic manifestations, imaging findings, associated malformations, treatment modalities, and outcomes—is missing.

Accordingly, the objective of this review is to gather and critically evaluate all published CTD cases to (1) delineate the range of clinical presentations and associated cardiac anomalies, (2) summarise diagnostic methods and imaging characteristics, (3) describe therapeutic approaches, whether conservative, percutaneous, or surgical, and their outcomes, and (4) identify gaps in current knowledge to inform future investigations and improve reporting standards. By consolidating scattered case reports and small series, this review aims at providing a comprehensive reference for clinicians and researchers, clarifying the existing understanding of CTD and highlighting directions for future study.

## 2. Search Methodology and Data Collection

A thorough literature search was carried out to identify all published reports of CTD up to October 2025. Searches were performed in the PubMed, MEDLINE, and Scopus databases using a combination of keywords and Medical Subject Headings (MeSH), including “cor triatriatum dexter”and “cor triatriatum dextrum”, and “persistent embryonic right valve of the sinus venosus.” Boolean operators were applied to enhance search sensitivity. Data extraction was independently performed by two reviewers (PPB and EF), with any discrepancies resolved through discussion with a third investigator (MAP). Additionally, reference lists of relevant publications were manually screened to capture any further eligible cases.

Inclusion criteria comprised case reports, case series, and observational studies explicitly describing CTD in human subjects. Reports not written in English, Spanish, French or Italian were excluded. Of note, several English-language cases were unavailable through libraries or corresponding authors. Foetal, autoptic, and animal cases were excluded as well. The review followed the PRISMA (Preferred Reporting Items for Systematic Reviews and Meta-Analyses) guidelines [18] (see Table S1, Supplementary Material).

Data extraction was conducted using a standardised form, capturing variables such as patient’s age, sex, presenting symptoms, electrocardiographic findings, chest radiograph features, echocardiographic data, CT and/or MRI results, presence of concomitant cardiac abnormalities, treatment approach (conservative, surgical, or device-based), and clinical outcomes. Descriptive statistics, including frequencies, percentages, means, and standard deviations for continuous variables, were computed.

## 3. Results

A search through the above stated libraries revealed a total of 114 case reports and case series on humans, either under the term “dexter” or “dextrum”, encompassing 124 patients. This final dataset was used for analysis [19,20,21,22,23,24,25,26,27,28,29,30,31,32,33,34,35,36,37,38,39,40,41,42,43,44,45,46,47,48,49,50,51,52,53,54,55,56,57,58,59,60,61,62,63,64,65,66,67,68,69,70,71,72,73,74,75,76,77,78,79,80,81,82,83,84,85,86,87,88,89,90,91,92,93,94,95,96,97,98,99,100,101,102,103,104,105,106,107,108,109,110,111,112,113,114,115,116,117,118,119,120,121,122,123,124,125,126,127,128,129,130,131,132,133] (see Table S2, Supplementary Material).

The mean age at presentation was 33.3 ± 9.4 years (age range 1 day–87 years). The sex ratio was close to parity. The patient’s sex was not reported in 4/124 (3.2%). Symptoms were reported in 122/124. The most frequent presenting symptom was dyspnoea (44.3%), followed by cyanosis (29.5%) and transient ischaemic accident/stroke (4.1%). Cyanosis occurred predominantly in neonates and infants, whereas dyspnoea was way more common in children and adults.

An ECG was performed in 75/124. Electrocardiographic findings demonstrated supraventricular tachycardia/atrial flutter/atrial fibrillation in 25/175 (33.3%) of CTD patients. Other electrocardiographic features were right bundle branch block (22.6%), right atrial enlargement (13.3%), and complete heart block (5.3%). A chest radiogram was done just in 43/124. On chest X ray, cardiomegaly was noted in 46.5%. Echocardiography served as the primary diagnostic modality in 95.2% of cases. Cardiac MRI and/or CT were utilised in 34.1% of cases, primarily to confirm the diagnosis of CTD. Specifically, MRI was used in 28/124 (22.6%), whilst CT was performed in 22/124 (17.7%).

Associated cardiac abnormalities were identified in 84/124 (67.7%), mainly as (all forms of) atrial septal defect (48/124; 38.7%) and associated right valvular and right ventricular involvement (tricuspid valve dysplasia, Ebstein’s anomaly, right ventricular hypoplasia, pulmonary valve stenosis, pulmonary valve atresia, right ventricular hypoplasia, right ventricular non compaction, arrhythmogenic right ventricular dysplasia, right ventricular outflow tract obstruction) in 20/124 (20.1%). 

An outcome was reported in 97/124. Surgical correction was the treatment of choice in 50/97 patients (51.5%). Since 1991, a percutaneous approach has been employed in selected cases (5/97; 5.1%). Conservative management was the treatment of choice in 42/97 (43.3%). Mortality rate of 8.2% (8/97).

The results of this study are summarised in Table 1.

## 4. Discussion

In a review by Doucette and Knoblich the most common location of this membrane was to the right of the superior vena cava, coronary sinus, and inferior vena cava and the second most common pattern found was when the membrane was to the left of the coronary sinus but to the right of the other two venous vessels [133].

Complete CTD remains an exceptionally rare congenital cardiac anomaly, and the present review highlights the broad clinical spectrum, diagnostic strategies, and management approaches that characterise this condition across the literature. Although CTD is traditionally regarded as a paediatric diagnosis because of its embryologic origin, the mean age at diagnosis in this review—33.3 years—demonstrates that it may remain clinically silent for decades or present incidentally in adulthood. The wide age range, spanning from infancy to the elderly, underscores the highly variable hemodynamic impact of the persistent right-sided membrane. For instance, CTD can be an important though rare cause of neonatal cyanosis in the presence of a concomitant right-to-left shunt through an atrial septal defect or a patent foramen ovale [134]. Cyanosis can present even in adulthood during exercise [135]. The degree of the shunt depends on the balance between the resistance of the membrane and left atrial pressure (i.e., the more the membrane is obstructive, the higher the degree of right-to-left shunting and consequently the more severe the symptoms) [136].

Sex ratio was well balanced [137]. More importantly, the clinical presentation was markedly heterogeneous, consistent with the degree of obstruction to right atrial flow and the presence of associated cardiac defects. Dyspnoea (43.6%) and cyanosis (29.4%) were the most frequent symptoms, reflecting impaired systemic venous return and right-sided inflow obstruction. These findings align with previous reports describing presentations that range from subtle exertional intolerance to profound hypoxemia, depending on membrane morphology and the presence of interatrial shunting.

Concerning CTD-related symptoms, they vary widely depending on how obstructive the membrane is and whether it affects blood flow from the venae cavae or to the tricuspid valve. If the CTD is isolated and the membrane is incomplete and non-obstructive, many people are asymptomatic, and the condition is found incidentally during surgery to correct other cardiac abnormalities or during imaging. They are defined as forme fruste CTD. Occasionally, they are incidentally detected in adults during transoesophageal echocardiography imaging procedures [138]. If the membrane is obstructive, it generally mimics symptoms of right-sided inflow obstruction or systemic venous congestion (lower-extremity oedema, abdominal fullness or discomfort, hepatomegaly, jugular venous distention, fatigue, especially with exertion, shortness of breath, palpitations (from associated atrial arrhythmias). Symptoms related to systemic venous obstruction include prominent neck veins and facial swelling (superior vena cava obstruction) as well as worsening peripheral oedema, ascites, cold, swollen lower limbs (inferior vena cava obstruction). Infants tend to show signs sooner if the defect is restrictive. They present with cyanosis (if there is associated right-to-left shunting), poor feeding, failure to thrive, tachypnoea, recurrent respiratory infections, hepatomegaly. Associated congenital abnormalities can add symptoms, such as cyanosis from right-to-left shunt, more prominent exercise intolerance, arrhythmias [139].

Electrocardiographic abnormalities, most notably supraventricular arrhythmias (20.1%) and right bundle branch block (13.5%), are likely secondary to chronic right atrial dilatation or altered atrial conduction pathways. However, their relatively modest prevalence reinforces that ECG findings are neither sensitive nor specific for CTD. Likewise, chest radiography showed cardiomegaly in only 15.9% of cases, further illustrating that routine non-invasive tests may appear normal despite significant intracardiac structural anomalies.

**Figure 1:**
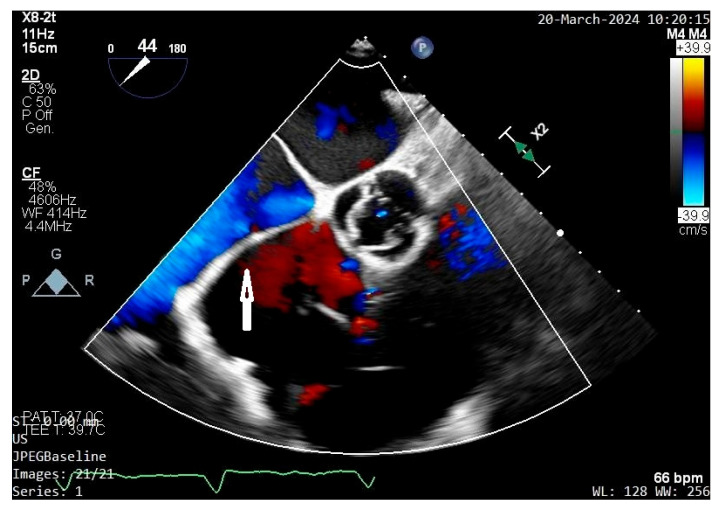
Transoeasophageal echocardiography displaying the cor triatriatum dexter membrane (white arrow) subdividing the right atrium into two chambers.

Echocardiography was the mainstay of diagnosis, identifying CTD in 95.2% of cases and proving indispensable in evaluating membrane morphology, right atrial flow dynamics, and associated anomalies. Since CTD subdivides the right atrium into two parts, even contrast echocardiography can be used to identify the membrane opening which, if restrictive, leads to delayed filling of the lower portion of the atrium [140]. See Figure 1.

**Figure 2:**
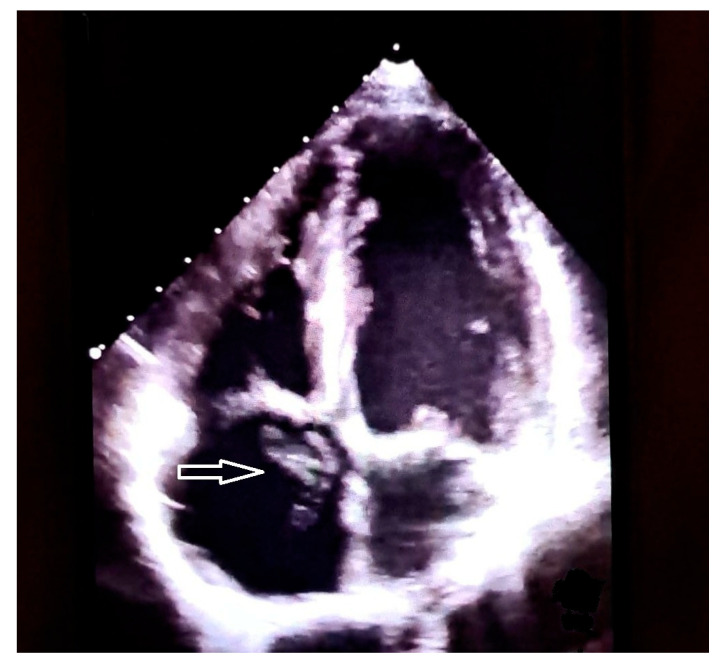
Transthoracic echocardiogram (4 chamber-view) showing a thin and mobile membrane floating in the right atrium (Chiari’s network, white arrow).

**Figure 3:**
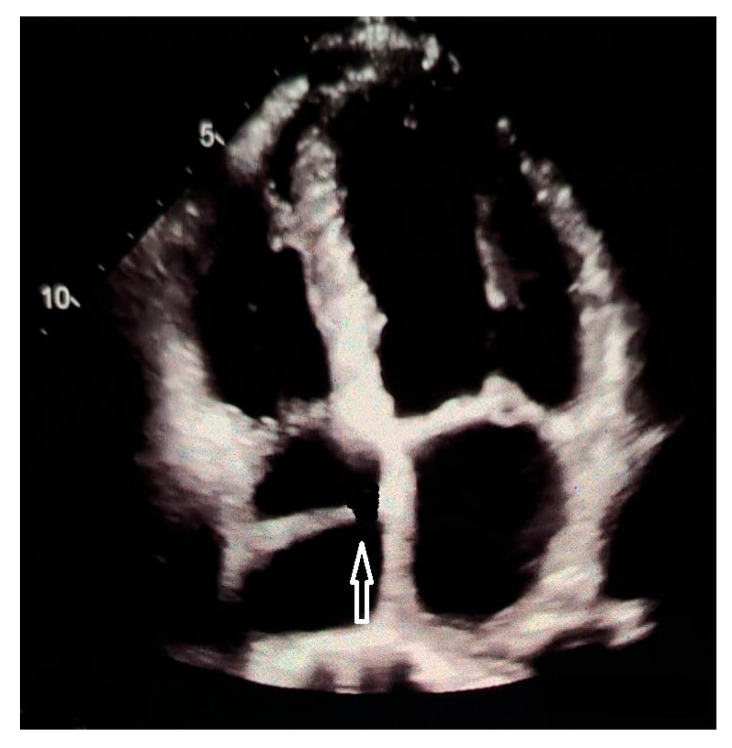
Transthoracic echocardiogram (4 chamber-view) showing a slightly mobile, linear, echogenic structure not reaching the interatrial septum (prominent Eustachian valve, white arrow).

**Figure 4:**
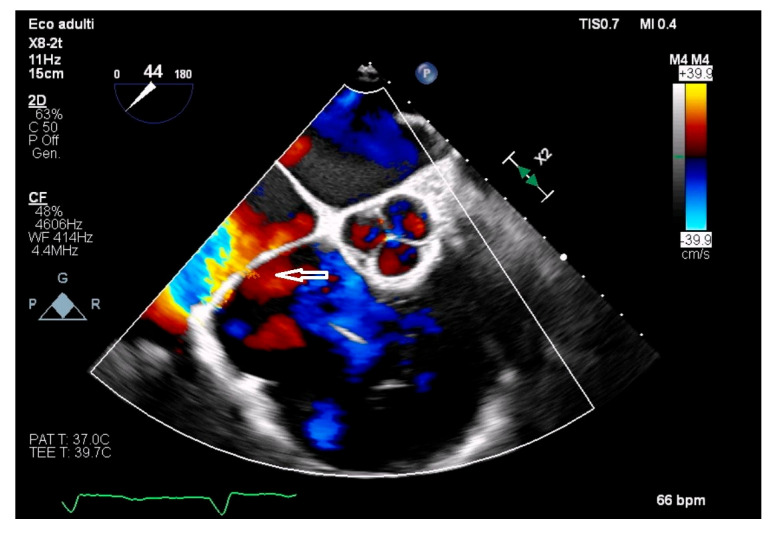
Transoesophageal echocardiogram displaying a membrane subdividing the right atrium into two chambers (cor triatriatum dexter. There is a mild turbulence across the membrane which is represented by a small orifice (white arrow).

In addition, three-Dimensional echocardiograpy may allow a precise reconstruction of the CTD membrane [141]. From an echocardiographic standpoint, there are 3 different types according to the location of the right atrial membrane. Differentiation between a giant Eustachian valve and CTD can be difficult. Although the embryologic explanation of CTD is the same as that of the normal formation of the Eustachian valve (lack of regression of the right sinus venosus valve), it is usually called CTD when there are attachments to the atrial septum, giving the appearance of a divided atrium. Conversely, it is called prominent Eustachian valve when the right sinus venosus valve is partly regressed, with no remaining septal attachments and without the appearance of a divided atrium. On echocardiography, the differential diagnosis must be established with the Chiari’s network as well, which has the echocardiographic appearance of a floating network of fibres, with multiple insertions in the upper part of the right atrium, and with the Eustachian valve located at the orifice of the inferior vena cava, with little mobility [142]. See Figure 2, Figure 3 and Figure 4 for differential diagnosis among Chiari’s network, prominent Eustachian valve and CTD.

Cross-sectional imaging using CT or MRI played a confirmatory role in one-third of patients, particularly when echocardiographic windows were limited or when further anatomical clarification was required for surgical or interventional planning [143]. These results reinforce current practice patterns, which favour echocardiography as the initial diagnostic modality while reserving advanced imaging for complex or ambiguous cases. Before the era of echocardiography, CTD could be diagnosed only by cardiac catheterization, heart surgery, or at autopsy [20]. Conversely nowadays CTD can be diagnosed by foetal echocardiography as well [144].

A particularly notable finding is the high prevalence (67.7%) of concomitant congenital heart defects. Atrial septal defects (38.1%) were most common, consistent with the shared embryologic origins and altered haemodynamics associated with right atrial partitioning. Right valvular and ventricular involvement (20.1%) further suggests that CTD often exists within a broader spectrum of right-sided congenital abnormalities rather than as an isolated anomaly [145]. The right valvular and right ventricular involvement might be due to the reduced blood flow from the septated right atrium towards the right ventricle. A unique combination of CTD and cor triatriatum sinister has been reported also [66]. The presence of associated defects has important implications for both symptom presentation and therapeutic decision-making.

Management strategies varied, reflecting the diverse anatomical and physiological presentations of CTD. In fact, the need for intervention depends on the number and the size of the fenestrations on the membrane, associated cardiac abnormalities and arrhythmias. Surgical resection of the dividing membrane remained the dominant approach (51.5%), particularly in symptomatic patients or those with significant associated cardiacanomalies. Since 1991, percutaneous interventions, such as percutaneous catheter disruption of the membrane or stent placement to enlarge the membrane orifice, have emerged as an alternative to open heart surgery in selected cases (5.1%), offering a less invasive option when membrane morphology is favourable. Incomplete CTD may hamper percutaneous atrial septal defect closure in about 5% of the cases. The presence of CTD is expected to render any percutaneous intervention that involves manipulation of catheters and devices into the right atrium very challenging (i.e., difficult catheter navigation or entrapment of the sheaths and/or the closure device) [146]. Catheter ablation is a feasible and efficient therapeutic strategy for treating complex atrial tachyarrhythmias in patients with CTD. Atrial remodelling due to anatomical obstruction or heterogeneous conduction of the fibromuscular membrane may serve as an arrhythmic substrate [88]. Hybrid intervention and endoscopic robotic correction have been performed as well [147,148]. Conservative management was chosen in approximately one third of cases, highlighting that asymptomatic or mildly symptomatic patients with minimal obstruction may be safely observed. In fact, asymptomatic patients are generally not treated unless they are undergoing cardiac surgery for other reasons. Importantly, the favourable overall outcomes—including a relatively low mortality rate of 8.2% mainly reported decades ago or due to concomitant cardiac abnormalities—suggest that timely diagnosis and carefully selected management strategies lead to excellent prognoses.

Overall, this review underscores that CTD, though rare, should be considered in the differential diagnosis of unexplained right-sided heart obstruction, cyanosis, or atypical venous return patterns, particularly when associated congenital defects are present. Advances in imaging and the evolution of interventional techniques continue to improve diagnostic accuracy and expand therapeutic options, contributing to increasingly favourable outcomes. Continued reporting of CTD cases will be essential to refining management strategies and enhancing understanding of this uncommon but clinically significant congenital anomaly.

This review has several limitations that should be considered when interpreting the findings. First, the analysis is based predominantly on case reports and small case series, reflecting the rarity of CTD. Such sources are inherently subject to publication bias, as atypical, symptomatic, or surgically treated cases are more likely to be reported, potentially overestimating the frequency of severe presentations and associated anomalies. Second, the heterogeneity of reporting across studies limited the ability to perform uniform comparisons. Key clinical details—such as membrane morphology, hemodynamic measurements, long-term follow-up, and indications for specific interventions—were inconsistently documented, reducing the precision of the analysis. Third, diagnostic and therapeutic practices have evolved over the decades covered by the review. Earlier reports relied heavily on invasive angiography or postmortem diagnosis, whereas more recent cases benefit from high-resolution imaging and improved interventional techniques. This temporal variability may have influenced both the diagnostic yield and treatment patterns observed. Fourth, outcome data were incompletely presented in several cases, which may affect the accuracy of the reported mortality rate and limit conclusions about long-term prognosis. Finally, because CTD often coexists with other congenital cardiac defects, isolating the contributions of CTD itself to symptoms, management decisions, and outcomes remains challenging. As a result, the clinical course described may not fully represent the natural history of isolated CTD. Despite these limitations, this review provides the most comprehensive synthesis to date and highlights important trends in the presentation, diagnosis, and management of this uncommon condition.

## 5. Conclusions

CTD is a rare and often under-recognised congenital anomaly with highly variable clinical manifestations. This review demonstrates that although many patients present with dyspnoea, cyanosis, or arrhythmias, others remain asymptomatic until adulthood. Echocardiography remains the cornerstone of diagnosis, frequently supplemented by CT or MRI for anatomical clarification. The high prevalence of associated congenital heart defects underscores the importance of comprehensive structural evaluation. Management strategies range from conservative observation to surgical or percutaneous intervention, depending on symptom burden and anatomical complexity. Overall outcomes are favourable when CTD is appropriately identified and treated. Continued case reporting and systematic data collection will help refine diagnostic approaches and guide management in this uncommon condition.

## Figures and Tables

**Table 1:** Cor triatriatum dexter patients’ main features.

Features	
Age	33.3 ± 9.4 years
Symptoms	dyspnoea 44.3% (54/122)
	Cyanosis 29.5% (36/122)
	TIA/stroke 4.1% (5/122)
ECG	SVT/AF/Afib 33.3% (25/75)
	RBBB 22.6% (17/75)
	RA enlargement 13.3% (10/75)
	CHB 35.3% (4/75)
Chest X-ray	Cardiomegaly 46.5% (20/43)
Echocardiography	used in 95.2% (118/124)
MRI	used in 22.6% (28/124)
CT	used in 17.7% (22/124)
Concomitant cardiac abnormalities	67.7% (84/124)
Treatment	Surgery 51.6% (50/97)
	Conservative 43.3% (42/97)
	Device-based 5.1% (5/97)
Death	8.2% (8/97)

Acronyms: TIA: transient ischaemic attack ECG: electrocardiogram; SVT: supraventricular tachycardia; AF: atrial flutter; Afib: atrial fibrillation; RBBB: right bundle branch block; RA: right atrium; CHB: complete heart block.

## Data Availability

No new data were created or analyzed in this study.

## Supplementary Materials

The following supporting information can be downloaded at https://www.mdpi.com/article/10.3390/jcdd13020076/s1, Table S1: PRISMA flow diagram; Table S2: the selected case reports and case series.
