# Supplementary material for: Cor Triatriatum Dexter: The Largest Comprehensive Review in the Field on 124 Worldwide Cases (1968–Now)
Source: J Cardiovasc Dev Dis. 2026 Feb 3;13(2):76. doi: 10.3390/jcdd13020076 (PMC12941072; doi:10.3390/jcdd13020076)
Supplement: Supplementary file 1 [file jcdd-13-00076-s001.zip › jcdd-4035213-supplementary.pdf]

Table S1. PRISMA Flow Diagram

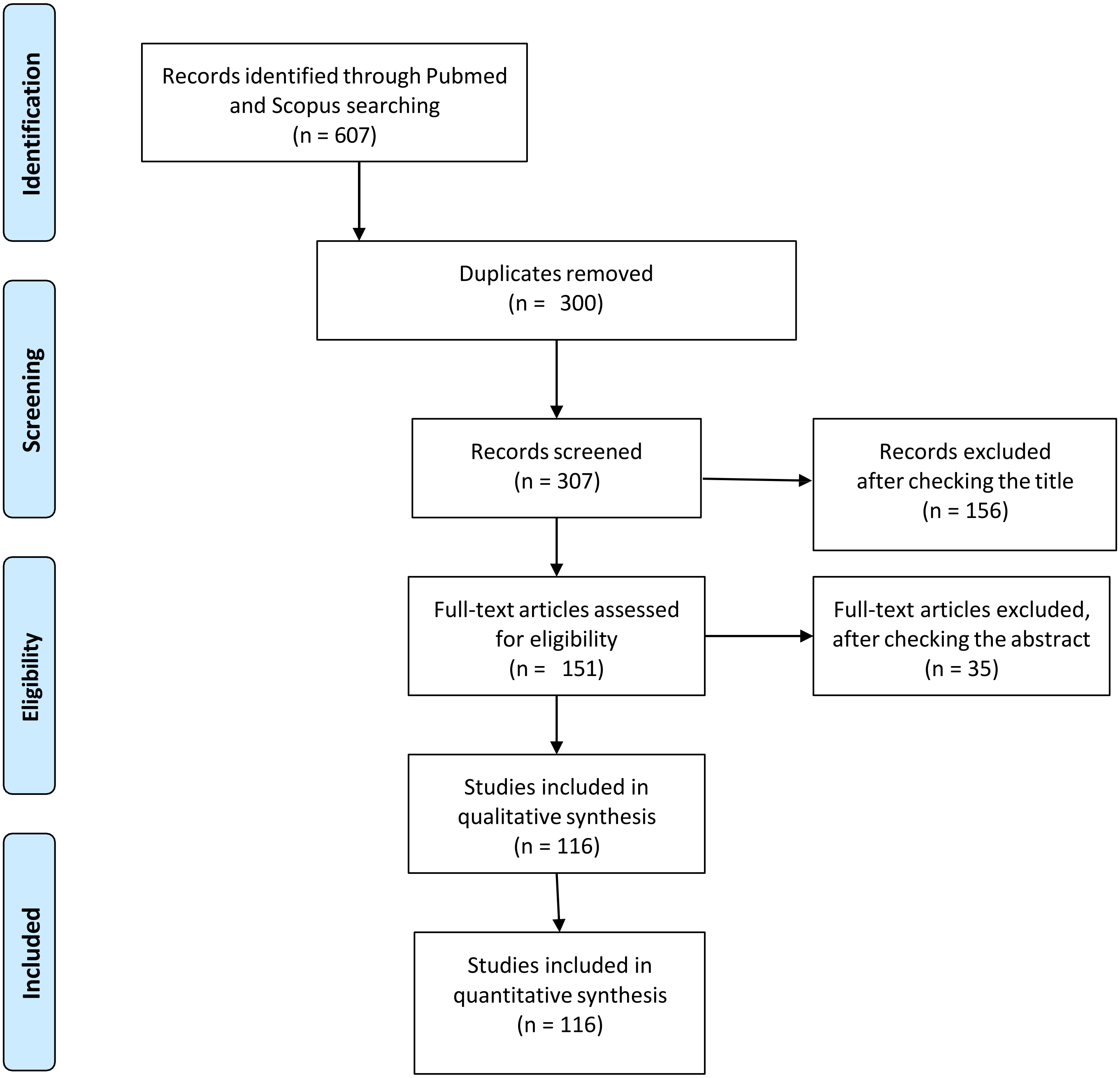

From: Moher D, Liberati A, Tetzlaff J, Altman DG, The PRISMA Group (2009). *P*referred *R*eporting *I*tems for *S*ystematic Reviews and *M*eta-*A*nalyses: The PRISMA Statement. PLoS Med 6(6): e1000097. doi:10.1371/journal.pmed1000097

For more information, visit www.prisma-statement.org.

Table S2. The selected case reports and case series

Reference	Age	Gender	Symptoms	ECG	X-ray	Echocardiography	MRI/CT	Associated cardiac anomalies	Outcome
Runcie J. **[19]**	26	Female	Dyspnoea, ascites	Low voltage complexes and flattened T waves	Minimal broadening of the right atrial mediatinum	Not available at that time	Not available at that time	None	Death
Hansing CE, et al. **[20]**	25	Male	Dyspnoea, syncope	Normal morphology	Not reported	Not available at that time	Not available at that time	Atrial septal defect	Surgery
Ott DA, et al. **[21]**	67	Female	Palpitations	Supraventricular tachycardia	Not reported	Not available at that time	Not available at that time	None	Surgery
Mazzucco A, et al. **[22]**	5 months	Male	Dyspnoea	Sinus rhythm	Pulmonary congestion	Not available at that time	Not available at that time	Anomalous systemic venous return, atrial septal defect	Surgery
Alboliras ET, et al. **[23]**	1 day	Female	Heart failure	Complete heart block	Not reported	Yes	No	Membranous and muscular ventricular septal defects, pulmonary stenosis	Death
Burton DA, et al. **[24]**	13 months	Male	Cyanosis	Low voltage in right precordial leads	Mild cardiomegaly	Yes	No	Atrial septal defect	Surgery
Trakhtenbroit A, et al. **[25]**	56	Female	Progressive cyanosis over the years, sudden onset of dyspnoea	Incomplete right bundle branch block	Normal	Yes	No	Ebstein's anomaly, atrial septal defect	Surgery
Savas V, et al. **[26]**	28	Female	Cirrhosis, cyanosis	Non specific T wave abnormality	Not reported	Yes	No	Hypoplastic right ventricle	Percutaneous transluminal dilatation
Adad SJ, et al. **[27]**	25	Male	Pulmonary thromboembolism	Sinus rhythm	Not reported	Yes	No	Pulmonary valve stenosis	Death
Fiorilli R, et al. **[28]**	56	Female	Dyspnoea	Incomplete right bundle block	Increased vascular markings	Yes	No	Sinus venosus atrial septal defect	Surgery
Dobbertin A, et al. **[29]**	47	Male	Cyanosis and dyspnoea, episodes of atrial flutter with heart failure	Incomplete right bundle branch block, atrial flutter	Mild cardiomegaly	Yes	No	Secundum atrial septal defect, persistent left superior vena cava	Surgery
Ebeid MR, et al. **[30]**	15 months	Female	Cianosys (Blalock-Taussig shunt)	Not reported	Not reported	Yes	No	Pulmonary atresia with	Conservative management,

								intact ventricular septum, atrial septal defect	atrial septal defect closure
	3	Male	Dyspnoea, cyanosis	Not reported	Not reported	Yes	No	Two atrial septal defects	Conservative management, atrial septal defect closure
Roldán FJ, et al. **[31]**	39	Male	Asymptomatic	Not reported	Not reported	Yes	No	Mechanical aortic and mitral valves due to rheumatic disease	Conservative management
Joe BN, et al. **[32]**	70	Male	Dyspnea, episode of congestive heart failure	Not reported	Not reported	Yes	Yes (MRI)	Atrial septal defect	Not reported
Bisinov EA, et al. **[33]**	30	Male	Hypotension	Not reported	Not reported	Yes	No	None	Death
Eroglu ST, et al. **[34]**	67	Male	Dysponea on exertion, palpitation, peripheral oedema	Atrial fibrillation, incomplete right bundle branch block	Cardiomegaly	Yes	No	Ebstein's anomaly, atrial septal defect	Conservative management
Kessel-Schaefer A, et al. **[35]**	33	Female	Bronchopneumonia, otherwise asymptomatic	Incomplete right bundle branch block	Cardiomegaly	Yes	Yes (MRI)	Sinus venosus atrial septal defect	Surgery
Caliskan M, et al. **[36]**	76	Male	Palpitation, dyspnoea on exertion, lower limb swelling	Atrial fibrillation, complete right bundle branch block	Cardiomegaly	Yes	No	Ebstein's anomaly	Conservative management
	32	Male	Dyspnoea on exertion	Incomplete right bundle branch block	Not reported	Yes	No	Small atrial septal defect	Conservative management
Steen H, et al. **[37]**	68	Not reported	Dyspnoea	Normal sinus rhythm	Not reported	Yes	Yes (MRI)	None	Not reported
Lee YS, et al. **[38]**	38	Female	Palpitation, chest discomfort	Left anterior fascicular block	Normal	Yes	No	None	Conservative management
	72	Female	Dyspnea on exertion, abdominal pain	Atrial fibrillation	Cardiomegaly, bilateral pleural effusion	Yes	No	None	Conservative management
Yarrabolu TR, et al. **[39]**	68	Female	Lymphedema and cellulitis of the lower left leg	Sinus rhythm	Cardiomegaly	Yes	No	None	Conservative management
Modi K, et al. **[40]**	67	Male	Lower limb oedema	Sinus tachycardia, left ventricular hypertrophy	Patchy opacities at the lung bases	Yes	No	None	Not reported
Galli MA, et al. **[41]**	2 days	Female	Severe central cyanosis	Not reported	Normal findings	Yes	No	Atrial septal defect	Surgery

Mohan JC, et al **[42]**	5	Male	Refractory heart failure	Not reported	Not reported	Yes	Yes (MRI)	Dysplastic pulmonary valve with severe regurgitation, perimembranous ventricular septal defect, mitral valve prolapse, diverticulum of the right ventricular outflow tract	Surgery
Barrea C, et al. **[43]**	4 months	Female	Cyanosis	Right atrial hypertrophy	Reduced pulmonary blood flow	Yes	No	None	Surgery
Hoye DJ, et al. **[44]**	5 days	Female	Cyanosis	Sinus bradycardia	Normal findings	Yes	No	None	Surgery
Maroules CD, et al. **[45]**	39	Female	Exertional dyspnoea	Not reported	Not reported	Yes	Yes (CT and MRI)	Secundum atrial septal defect	Surgery
Sánchez-Brotons JA, et al. **[46]**	76	Female	Dyspnoea	Not reported	Not reported	Yes	No	None	Not reported
	68	Female	Asymptomatic	Not reported	Not reported	Yes	No	None	Not reported
Januszewska K, et al. **[47]**	1 day	Male	Central cyanosis	Not reported	Not reported	Yes	No	Secundum atrial septal defect	Surgery
Salam S, et al. **[48]**	4 days	Female	Cyanosis	Right atrial hypertrophy	Cardiomegaly	Yes	No	Secundum atrial septal defect	Surgery
Cartón AJ, et al. **[49]**	2 days	Male	Cyanosis	Not reported	Relative pulmonary ischaemia	Yes	No	None	Surgery
Fesslova V, et al. **[50]**	1 day	Male	Cyanosis	Not reported	Not reported	Yes	No	Hypoplastic right ventricle	Surgery
Martínez-Quintana E, et al. **[51]**	44	Male	Dyspnoea on exertion	Atrial fubrillation	Not reported	Yes	Yes (CT)	Secundum atrial septal defect	Surgery
Zainudin AR, et al. **[52]**	9	Male	Low saturation (88-92%)	Not reported	Not reported	Yes	No	Secundum atrial septal defect	Surgery
Udovičić M, et al. **[53]**	48	Male	Spastic quadriplegia, cyanosis	Right ventricular hypertrophy	Not reported	Yes	No	Tetralogy of Fallot	Death
Choudhary D, et al. **[54]**	19	Male	Dyspnoea on exertion	Right atrial enlargement, incomplete right bundle branch block	Cardiomegaly	Yes	Yes (MRI)	None	Conservative management
Aldawoodi NN, et al. **[55]**	30	Female	End stage renal disease	Not reported	Not reported	Yes	Yes (CT)	None	Death

Nageh MF, et al. **[56]**	59	Male	Palpitations	Typical atrial flutter	Not reported	Yes	Yes (CT and MRI)	Ventricular septal defect	Ablation. Otherwise not reported
Yamaguchi R, et al. **[57]**	82	Female	Palpitations, chest oppression, exertional dyspnoea	Atrial fibrillation	Cardiomegaly, vascular congestion	Yes	Yes (CT)	Secundum atrial septal defect	Conservative management
Tufaro V, et al. **[58]**	63	Female	Acute heart failure	Not reported	Not reported	Yes	Yes (CT)	None	Death
Low TT, et al. **[59]**	50	Male	Congestive heart failure	Atrial flutter with variable conduction	Not reported	Yes	No	None	Conservative management
Qureshi AU, et al. **[60]**	2 weeks	Female	Cyanosis	Sinus rhythm	Normal	Yes	No	Secundum atrial septal defect, patency of ductus arteriosus	Surgery
Guler Y, et al. **[61]**	32	Female	Exertional dyspnoea	Complete atrio-ventricular block	Normale	Yes	Yes (CT and MRI)	None	Permanent pacemaker insertion, otherwise conservative management
Omeje I, et al. **[62]**	7	Female	High blood pressure, intermittent leg pains, and diminished femoral pulses	Not reported	Not reported	Yes	Yes (MRI)	Aortic coarctation, severe narrowing of the inferior vena cava and venous return via the azygos and hemiazygos veins to the superior vena cava and the brachiocephalic veins, respectively	Surgery and aortic stenting
Simsek Z, et al. **[63]**	22	Female	Dyspnoea, palpitations, near syncope	Atrial fibrillation, complete right bundle branch block, premature ventricular complexes	Cardiomegaly	Yes	Yes (CT)	Primum and secundum atrial septal defects, pulmonary stenosis	Not reported
Yerebakan C, et al. **[64]**	1 week	Female	Cyanosis	Not reported	Not reported	Yes	Yes (MRI)	Atrial septal defect	Surgery
Kilit C, et al. **[65]**	58	Male	Dyspnoea, palpitations, lower limb oedema	Atrial fibrillation	Not reported	Yes	No	Pectus excavatum	Conservative management

Al-Mousily F, et al **[66]**	11 months	Female	Abdominal distention and facial swelling	Not reported	Lung congestion and bilateral pleural effusion	Yes	Yes (MRI)	Cor triatriatum sinister, right upper pulmonary vein stenosis	Surgery
Vukovic PM, et al. **[67]**	43	Female	Dyspnoea on exertion	Not reported	Not reported	Yes	No	Atrial septal defect	Disruption of the membrane by percutaneous balloon dilation then surgical closure of the atrial septal defect
Mackman CA, et al. **[68]**	1 day	Female	Desaturation	Not reported	Not reported	Yes	No	Atrial septal defect	Surgery
	1 day	Female	Desaturation	Not reported	Normal	Yes	No	None	Surgery
	1 day	Female	Dysmorphic, feeding difficulties	Not reported	Not reported	Yes		Cornelia de Lange Syndrome	Surgery
Hwang SH, et al. **[69]**	56	Male	Palpitations, dyspnoea	Atrial fibrillation	Not reported	Yes	Yes (MRI)	Gian Eustachian valve	Not reported
Hussain ST, et al. **[70]**	22	Female	Acute-onset left sided chest pain and shortness of breath due to pulmonary embolism	Not reported	Not reported	Yes	No	Atrial septal defect	Surgery
Özmen G, et al. **[71]**	19	Female	Atypical chest pain	Sinus rhythm	Not reported	Yes	No	Mitral valve cyst, atrial septal aneurysm	Not reported
Eckersley LG, et al. **[72]**	14	Male	Desaturation on exertion, poor exercise tolerance	Not reported	Not reported	Yes	Yes (CT and MRI)	Atrial septal defect, mild hypoplasia of the right ventricle	Atrial septal defect device closure, otherwise conservative management
Alghamdi MH. **[73]**	1 week	Female	Desaturation and cyanosis	Sinus rhythm	Normal	Yes	No	None	Unsuccessful percutaneous procedure, then surgery
Elagizi A, et al. **[74]**	40	Male	Palpitations, and lower extremity edema.	Atrial fibrillation	Not reported	Yes	No	Persistent left superior vena cava with an unroofed coronary sinus, coronary fistula between the	Conservative management

								right coronary artery and right atrium	
Anyanwu LJ, et al. **[75]**	16 months	Male	Dysmorphic	Sinus rhythm	Not reported	Yes	No	Schinzel-Giedion syndrome	Conservative management
Sozzi FB, et al. **[76]**	23	Female	Asymptomatic	Sinus rhythm	Normal	Yes	Yes (MRI)	Atrial septal defect, pulmonary stenosis	Conservative management
Bennett JM, et al. **[77]**	84	Male	Worsening lower limb oedema	Not reported	Not reported	Yes	Yes (CT)	Severe tricuspid valve regurgitation	Unsuccessful percutaneous procedure for tricuspid valve regurgitation
León RL, et al. **[78]**	1 day	Female	Cyanosis, desaturation	Bradycardia	Not reported	Yes	No	None	Stroke
Zoltowska D, et al. **[79]**	75	Male	Asymptomatic	Atrial fibrillation	Not reported	Yes	No	None	Conservative management
Aliyu I, et al. **[80]**	3 months	Female	Difficulty with breathing and central cyanosis, poor weight gain	Biventricular hypertrophy	Cardiomegaly	Yes	No	Transposition of the great vessels, atrial septal defect, ventricular septal defetc	Death
Theodoropoulos KC, et al. **[81].**	54	Male	Embolic cerebellar infarcts	Not reported	Not reported	Yes	Yes (MRI)	None	Conservative management
Rao S, et al. **[82]**	10 months	Male	Cyanosis	Not reported	Not reported	Yes	Yes (MRI)	Pulmonary stenosis, mild right ventricular hypoplasia, secundum atrial septal defect	Surgery
Rozema TK, et al. **[83]**	1 day	Female	Cyanosis and hypotension	Not reported	Not reported	Yes	No	Cor triatriatum sinister, separate drainage of a left hepatic vein to the posterior aspect of the right atrium, common atrium	Surgery
Haboub M, et al. **[84]**	3	Male	Dyspnoea on moderate exertion	Right atrial enlargement, negative T waves in DI and aVL	Cardiomegaly, clearly decreased pulmonary vascular markings	Yes	No	Subpulmonary stenosis	Balloon valvuloplasty of subpulmonic membrane, conservative

									management of cor triatrum dexter
Morita Y, et al. **[85]**	36	Female	Fatigue	Not reported	Mild cardiomegaly with mild pulmonary congestion	Yes	Yes (CT and MRI)	Primum atrial septal defect, mitral valve prolapse	Surgery
Bindra BS, et al. **[86]**	38	Female	Chest pain	Q-wave changes in V1/V2	Not reported	Yes	No	None	Conservative management
Tzeis S, et al. **[87]**	62	Male	Palpitations, dyspnoea	Atrial fibrillation	Not reported	Yes	Yes (MRI)	None	Ablation, conservative management as to cor triatriatum dexter
Lugtu IC, et al. **[88]**	49	Male	Palpitations, dyspnoea	Atrial fibrillation	Not reported	Yes	Yes (CT)	None	Ablation, conservative management as to cor triatriatum dexter
Hurtado-Sierra D, et al. **[89]**	18 days	Not reported	Intermittent cyanosis	Not reported	Normal	Yes	No	Systemic venous flow partial obstruction	Not reported
Alvarez-Santana R, et al. **[90]**	23	Female	Dyspnoea, palpitations, cyanosis	Atrial fibrillation, right bundle branch block	Not reported	Yes	Yes (MRI)	Ebstein's anomaly, secundum atrial septal defect	Conservative management
Goel A, et al. **[91]**	35	Female	Not reported	Not reported	Not reported	Not reported	Yes (CT)	Sinus venosus and secundum atrial septal defects, partial anomalous pulmonary venous frainage	Surgery
Minciunescu A, et al. **[92]**	21	Male	Seizures and brain abscess	Atrial enlargement and incomplete right bundle branch block	Not reported	Yes	Yes (MRI)	Ebstein's anomaly, secundum atrial septal defect	Neurosurgery, then loss of follow-up
Kalangos A, et al. **[93]**	5	Female	Dyspnoea, palpitations	Sinus rhythm	Not reported	Yes	No	None	Surgery
Bhandari M, et al. **[94]**	30	Male	Syncope	Complete heart block	Not reported	Yes	No	Secundum atrial septal defect, right ventricular non compation	Surgery, permanent pacemaker insertion

Derimay F, et al. **[95]**	84	Female	Dyspnoea and severe chronic right cardiac failure	Ventricular paced rhythm (permanent pacemaker)	Not reported	Yes	Yes (CT)	Interatrial septal aneurysm	Percutaneous correction with stent implantation
Persia-Paulino YR, et al. **[96]**	49	Not reported	Transient ischaemic attack	Sinus rhythm	Not reported	Yes	No	None	Conservative management
Hanna G, et al. **[97]**	70	Male	Dyspnoea on exertion and fatigue	Not reported	Not reported	Yes	No	Interatrial septal aneurysm	Conservative management
Barbieri F, et al. **[98]**	78	Female	Dyspnoea and right cardiac failure	Atrial fibrillation	Not reported	Yes	Yes (CT)	Severe tricuspid regurgitation)	Percutaneous clipping of the tricuspid valve, otherwise conservative management
Poretti G, et al. **[99]**	3 days	Not reported	Cyanosis	Not reported	Not reported	Yes	No	Total anomalous pulmonary venous drainage	Surgery
Patel M, et al. **[100]**	36	Female	Palpitations, fatigue	Atrial fibrillation	Cardiomegaly	Yes	Yes (CT)	Right atrial appendage aneurysm	Surgery
Sunthankar S, et al. **[101]**	6 weeks	Male	Cyanosis	Not reported	Not reported	Yes	No	Hypoplastic right ventricle	Surgery
	1 day	Female	Cyanosis	Not reported	Not reported	Yes	No	None	Surgery
	1 day	Female	Cyanosis	Not reported	Not reported	Yes	No	Mild tricuspid stenosis	Conservative management
	1 day	Male	Cyanosis	Not reported	Not reported	Yes	No	Pulmonary atresia with intact ventricular septum	Surgery
Hasnie UA, et al. **[102]**	54	Female	Shortness of breath on exertion, worsening exercise tolerance	Sinus rhythm	Not reported	Yes	Yes (CT)	Atrial septal defect, ventricular septal defect	Percutaneous balloon dilation of the cor triatriatum dexter and insertion of a closure device for the atrial septal defect
Chen PH, et al. **[103]**	55	Female	Chest discomfort	Incomplete right bundle branch block	Right ventricle hypertrophy with prominent pulmonary congestion	Yes	Yes (CT)	Two secundum atrial septal defects	Atrial septal defect complicated by transient heart ischaemia

									due to coronary sinus obstruction, otherwise conservative management
De Michele F, et al. **[104]**	68	Female	Palpitations	Ventricular ectopic beats	Not reported	Yes	Yes (MRI)	None	Conservative management
Fuentes Rojas SC, et al. **[105]**	70	Female	Dyspnoea on exertion and lower extremity oedema	Atrial fibrillation and sick sinus syndrome status post dual-chamber pacemaker	Not reported	Yes	Yes (CT and MRI)	Torrential tricuspid valve regurgitation	Tricuspid valve surgery, otherwise conservative management
Picciolli I, et al. **[106]**	1 day	Male	Cyanosis	Sinus rhythm and normal morphology	Normal	Yes	No	Hypoplastic right ventricle	Surgery
Liang L, et al. **[107]**	25	Female	Chest discomfort, fatigue and syncope following intense physical activity	Sinus rhythm with P-wave abnormalities, incomplete right bundle branch block and right ventricular hypertrophy	Not reported	Yes	Yes (CT and MRI)	Hypertrophic cardiomyopathy	Not reported
Caputo A, et al. **[108]**	1 day	Male	Cyanosis	Sinus rhythm	Not reported	Yes	No	None	Surgery
He L, et al. **[109]**	49	Female	Migraine	Not reported	Not reported	Yes	No	Inferior sinus venosus and secundum atrial septal defect	Percutaneous intervention
Anastasakis E, et al. **[110]**	56	Male	Exertional dyspnoea and chest pain	Not reported	Not reported	Yes	No	Bicuspid aortic valve	Bioprosthetic aortic valve, otherwise conservative management
Binder MS, et al. **[111]**	51	Female	Exertional dyspnoea	Not reported	Not reported	Yes	No	Dysplastic tricuspid valve with severe regurgitation	Not reported
Kazma H, et al. **[112]**	50	Female	Dyspnoea, orthopnoea, and palpitations	Atrial fibrillation and incomplete right bundle branch block	Cardiomegaly	Yes	No	Sinus venosus atrial septal defect	Not reported
Roldan CA, et al. **[113]**	42	Female	Lower limb oedema and dizziness	Right atrial enlargement and rightward axis	Normal	Yes	No	None	Surgery

				deviation, and poor R-wave progression					
Kuźma J, et al. **[114]**	6 days	Male	Cyanosis, tachycardia, and tachypnoea	Supraventricular tachycardia	Not reported	Yes	No	Ventricular septal defect, aortic atresia, hypoplastic aortic artery and aortic arch	Surgery
	21 days	Female	Mild cyanosis and irregular heart beats	Premature atrial beats	Not reported	Yes	No	None	Surgery
Hernández-Benítez R, et al. **[115]**	7 days	Male	Cyanosis	Sinus rhythm	Normal	Yes	Yes (MRI)	Atrial septal defect	Surgery
Alotay A, et al. **[116]**	7 years	Female	Cyanosis	Not reported	Not reported	Yes	Yes (MRI)	Atrial septal defect	Percutaneous intervention
Daralammouri Y, et al. **[117]**	70	Female	Asymptomatic (cardiac examination required by oncology)	Not reported	Not reported	Yes	No	None	Conservative management
Shah B, et al. **[118]**	25	Male	Exertional dyspnoea and fatigue	Complete right bundle branch block	Right atrial enlargement with prominent pulmonary arteries	Yes	Yes (CT)	Secundum atrial septal defect	Surgery
Killen AW, et al. **[119]**	9	Female	Dyspnoea on exertion, palpitations	Right-axis deviation, enlargement of the right atrium, and right ventricular hypertrophy	Prominent right heart border	Yes		Secundum atrial septal defect, severe pulmonary valve stenosis	Surgery
Wang J, et al. **[120]**	51	Female	Dizziness, amaurosis, right limb numbness.	Sinus bradycardia (sick sinus syndrome)	Not reported	Yes	Yes (CT)	Absent inferior vena cava, polysplenia syndrome	Permanent pacemaker insertion
Taha AK, et al. **[121]**	45	Female	Progressive dyspnoea, fatigue, and lower limb edema	Sinus tachycardia	Not reported	Yes	No	None	Surgery
Franco E, et al. **[122]**	74	Male	Right sided heart failure symptoms	Paced rhythm (permanent pacemaker)	Not reported	Yes	No	Arrhythmogenic right ventricular cardiomyopathy	Conservative management
Chaaban B, et al. **[123]**	45	Female	Exertional dyspnoea, peripheral 0edema, hepatomegaly, and severe bilateral varicose veins	Not reported	Not reported	Yes	No	None	Conservative management

Thomka I, et al. **[124]**	7	Female	Fatigue, breathlessness on exertion and slight cyanosis	Right ventricular hypertrophy	Cardiomegaly	Yes	No	Secundum atrial septal defect	Surgery
Lepage JR. et al. **[125]**	46	Female	Anasarca, ascites, dyspnoea	Massive right atrial hypertrophy., abnormal ST-T waves with prolonged QT interval	Not reported	Not available at the time	Not available at the time	None	Surgery
Sarikouch S, et al. **[126]**	43	Male	Dyspnoea on exertion	Normal morphology	Not reported	Yes	MRI	Atrial septal defect, mild pulmonary stenosis	Surgery
Sahin T, et al. **[127]**	19	Male	Respiratory distress and fatigue	Right axis deviation, incomplete right bundle branch block, P pulmonale, right ventricle hypertrophy and negative T waves between V1–V5 derivatives	Mild cardiomegaly	Yes		Infundibular-valvar pulmonary stenosis	Surgery, but conservative management as to cor triatriatum dexter
Benyounes N, et al. **[128]**	64	Male	Stroke	Not reported	Not reported	Yes	Not reported	None	Not reported
Al-Mousily F, et al. **[129]**	11 months	Female	Abdominal distention and facial swelling	Not reported	Lung congestion and bilateral pleural effusion	Yes	Yes (MRI)	Pulmonary vein stenosis	Surgery
Xiang K, et al. **[130]**	87	Female	Dyspnoea on exertion	Atrial fibrillation, high grade atrioventricular block	Not reported	Yes	No	Atrial septal defect	Permanent pacemaker insertion
Montealegre-Gallegos M, et al. **[131]**	47	Male	Not reported	Not reported	Not reported	Yes	No	Atrial septal defect, ventricular septal defect, quadricuspid aortic valve	Surgery, but conservative management as to cor triatriatum dexter
Aboukhoudir F, et al. **[132]**	59	Male	Transient ischaemic accident	Atrial fibrillation	Cardiomegaly	Yes	Yes (MRI)	None	Conservative management
